# Measuring student-perceived cultural sustainability competence in guzheng education: development and preliminary validation of the GCSCS

**DOI:** 10.3389/fpsyg.2026.1900263

**Published:** 2026-10-28

**Authors:** Nina Liu, Yumeng Wu, Jun Jiang

**Affiliations:** 1 School of Education Science, Harbin Normal University, Harbin, China; 2 School of Preschool Education, Harbin Preschool Teachers College, Harbin, China; 3 Heilongjiang University, Harbin, China

**Keywords:** Chinese traditional music, cultural sustainability competence, digital communication, exploratory factor analysis, guzheng education, heritage identity, higher arts education, learner profiles

## Abstract

**Introduction:**

Cultural sustainability in guzheng education involves heritage understanding, embodied musical practice, creative adaptation, digital communication, and community participation. This study developed and preliminarily evaluated the Guzheng Cultural Sustainability Competence Scale (GCSCS) as a domain-specific self-report measure of students' perceived understanding, capability, judgment, and readiness.

**Methods:**

The initial 20-item scale was reviewed by five experts and administered to 168 guzheng students from five higher education institutions in Northeast China. Item screening, exploratory factor analysis, reliability analysis, descriptive statistics, and exploratory cluster analysis were conducted.

**Results:**

Two items were removed because their factor-loading patterns did not clearly correspond to their intended dimensions. The final 18-item scale showed good sampling adequacy (KMO = 0.882), a significant Bartlett's test of sphericity (χ^2^(153) = 1228.887, *p* < 0.001), and an interpretable five-factor solution explaining 53.35% of the total variance. Internal consistency was acceptable to good across the five subscales (Cronbach's α = 0.752–0.846 and McDonald's ω = 0.757–0.846). Heritage identity and embodied performance literacy received relatively higher self-reported scores than digital communication and community-oriented dimensions. Exploratory cluster analysis yielded four descriptive learner-profile patterns, although cluster separation was modest.

**Discussion:**

The findings provide preliminary psychometric evidence for examining multidimensional student self-perceptions in guzheng education. The results should not be interpreted as evidence of objectively demonstrated competence, stable learner categories, or causal effects of educational practices. Further validation in independent samples is needed.

## 1 Introduction

University music education is often evaluated through visible outcomes such as performance accuracy, repertoire difficulty, competition results, and stage experience. These outcomes are important, especially in professional training programs for traditional instruments. However, traditional music learning also involves less visible forms of development, including cultural understanding, aesthetic judgment, identity formation, communication ability, and students' perceived responsibility for cultural transmission. For students of the guzheng, a Chinese zheng zither with a long history and an expanding contemporary performance culture, learning the instrument is therefore not only a matter of technical progress. It also involves learning how to understand, interpret, communicate, and adapt a culturally embedded musical tradition.

This broader view of music learning can also be understood as a question of student-perceived competence. In educational psychology, student learning is not assessed only through external performance outcomes, but also through how learners perceive their knowledge, abilities, responsibilities, and readiness for future learning or participation. In traditional music education, this perspective is especially relevant because students are expected to develop not only technical performance ability, but also cultural understanding, aesthetic judgment, communication readiness, and a sense of responsibility toward cultural transmission. Therefore, a measurement approach is needed to examine how students perceive these competence areas in a specific learning context.

Cultural sustainability is one approach to putting a frame on this question. Preservation of intangible cultural heritage is not only dependent on artifacts or fixed repertoires, but also on active practice, interpretation, participation and intergenerational transmission (UNESCO, 2003; Hawkes, 2001; United Cities and Local Governments, 2010; Soini and Birkeland, 2014; Dessein et al., 2015; Smith and Akagawa, 2009). Culture has also been defined as a crucial aspect of sustainable development as it influences how communities perceive meaning, identity, creativity, and social belonging (UNESCO, 2019; Hawkes, 2001; United Cities and Local Governments, 2010; Soini and Birkeland, 2014; Dessein et al., 2015; Smith and Akagawa, 2009). This implies that in the context of traditional music education, the students are not supposed to just reproduce musical techniques but also have the chances to comprehend the cultural implications of the repertoire, convey these implications to other people, and come up with a well-informed decision regarding how tradition can be sustained in modern conditions.

The advent of digital communication has rendered this work more complicated. Digital media, short clips, live streaming, digital libraries, and interactive media can increase the availability of conventional music and provide fresh opportunities of cultural engagement (Yu, 2023; Cai et al., 2024). The studies of Chinese traditional instrumental culture have also demonstrated that interactive and digital methods may be used to communicate traditional culture when they are integrated with cultural significance instead of being viewed as mere technical devices (Shen et al., 2022). Meanwhile, digital distribution can make aesthetic expressions easier or take music activities out of the historical and cultural setting. Hence, digital literacy of the students must not be taken to mean merely being able to use online platforms. It must also involve being able to represent guzheng culture in a precise, significant and responsible manner to various audiences.

The guzheng can be considered as a valuable framework to discuss these matters. Its contemporary evolution is an expression of the relationship between the history of traditional practice, conservatory teaching, growth of a repertoire, stage presentation, teaching, and cultural identity (Sun, 2015; Silverberg, 2022; Han, 2013). The university guzheng programs are very significant in the formation of performers, instructors and future cultural professionals. However, the educational objectives of learning guzheng are not limited to performance skills alone. It is anticipated that students will appreciate the cultural significance of the instrument, acquire the bodily and aesthetic literacy, react to the current repertoire and adaptation, engage the audience at large, and explore some potential career options on the basis of teaching, performance, communication in culture, and heritage.

The cultural experience, identity, multicultural education, culturally responsive instruction, and the sustainability of traditional music education have been considered by previous studies of Chinese music education (Lau, 2008; Schippers, 2010; Elliott and Silverman, 2015; Du and Leung, 2022; Guan et al., 2023; Yang and Welch, 2023) and more recent research on guzheng music literacy has also underlined the fact that guzheng education cannot be considered solely as technical training, but it must also promote cultural heritage (Huang et al., 2025). Other research on the use of digital or online guzheng learning also indicates that digital environments can facilitate professional and intercultural competence as long as they are based on proper pedagogical design (Yin, 2023). Although this is an emerging body of literature, there remains a measurement gap. The talks about guzheng education tend to be conceptual, historical, and pedagogical; however, there are very few tools to measure the perceived cultural sustainability competence of students in a systematic manner.

The gap is important as it implies that curriculum reform and the support of the learner cannot be based on mere assumptions regarding cultural value. Teachers should be able to determine what competence areas are well-developed and which ones are poorly developed. They also need to find out if students have various profiles of learners. As an instance, certain students might have a high level of performance literacy and a lower level of digital communication preparedness, whereas other students could be more innovative and not as traditional in terms of repertoire and aesthetics. The development of scales offers a means of converting general educational constructs into quantifiable factors (Boateng et al., 2018; DeVellis and Thorpe, 2021). Exploratory factor analysis and reliability tests may provide preliminary clues on whether theoretically derived dimensions have empirical validity (Costello and Osborne, 2005; Hair et al., 2019). The cluster analysis can also detect clusters of learners with a varied pattern of competence and educational requirements (Kaufman and Rousseeuw, 1990).

This gap was filled by the development and preliminary validation of the Guzheng Cultural Sustainability Competence Scale (GCSCS) in the current study amongst the university students studying guzheng. It was intended to assess self-perceived student competence in five theoretically-based dimensions namely heritage identity and cultural responsibility; embodied performance and aesthetic literacy; creative adaptation and repertoire innovation; digital transmission and public communication; and community engagement and sustainable career preparedness. The sample consisted of 168 guzheng students at five higher education institutions in the Northeastern China region. The goal was not to give a national representation of every guzheng student in China, but to give initial psychometric support to a domain-specific tool in higher arts education.

The study addressed the following research questions:

RQ1: What dimensions constitute guzheng cultural sustainability competence among university guzheng students?
RQ2: Does the proposed GCSCS show acceptable preliminary factor structure and internal consistency?
RQ3: What patterns of perceived competence are observed across the five retained dimensions?
RQ4: What learner profiles can be identified based on GCSCS subscale scores?

These questions provide three contributions to the study. Firstly, the study conceptualizes cultural sustainability competence of guzheng as multidimensional self-perceived competence construct within a culturally embedded music education environment. Secondly, it gives preliminary psychometric evidence of the GCSCS being a domain-specific measurement instrument. Finally, it employs the analysis of learner profiles in order to reveal the differences between guzheng learners with regard to heritage identity, embodied performance literacy, innovation readiness, digital communication, and community participation. Such results can be useful in further studies of traditional music education, cultural competence evaluation, and individualized educational assistance in higher education in arts.

## 2 Theoretical background and conceptual framework

### 2.1 Cultural sustainability as a context for competence measurement

Sustainability can no longer be considered as the limited concept of environmental preservation; it has been extended to cover social responsibility, cultural continuity, educational transformation and competence development of the future. This change in higher education has seen universities being pushed to think of discipline-based knowledge, as well as other general abilities that students are required to have to understand, talk about, and put into use what they have learned in social and cultural contexts (United Nations, 2015; UNESCO, 2017). Sustainable Development Education places greater emphasis on values, involvement, responsibility and action-oriented learning as opposed to mere transfer of knowledge (UNESCO, 2020). In the case of arts education, it implies that professional training must be associated with cultural interpretation, awareness of values, social involvement, and the preservation of artistic tradition.

The concept of culture has also gained more attention as one of the key elements of sustainability. According to previous literature, culture was considered as the fourth pillar of sustainable development in addition to economic, social, and environmental aspects (Hawkes, 2001; United Cities and Local Governments, 2010). Subsequently, other articles elaborated on culture by defining it as being both in and out of sustainable development, as well as being sustainable development, indicating that culture is not merely a development resource, but also a mode of interpreting values, meanings, identities, and social practices (Soini and Birkeland, 2014; Dessein et al., 2015). Those views are especially applicable to the field of traditional musical education. Here culture is not something that can be used as an additional element to technical training. Culture determines the meanings, practices, identities and responsibilities in the context of music learning.

The idea of intangible cultural heritage is a significant background to the current research. According to the UNESCO Convention on the Safeguarding of the Intangible Cultural Heritage, it is the practices, representations, expressions, knowledge and skills which are deemed by communities to be part of their cultural heritage (UNESCO, 2003). In contrast to material heritage, intangible heritage is much more dependent on sustained practice, interpretation, involvement, and transmission. Traditional music education in universities should then not be measured simply in regard to student ability to re-create technique or play set repertoire. Instead, it needs to take into account whether students have the ability to comprehend, decipher, express, and act upon cultural implications that exist in musical practice.

The UNESCO Framework of Culture and Arts Education also indicates that arts education has an important role in cultural diversity, creativity, lifelong learning, digital transformation, and sustainable development (UNESCO, 2024). In this sense, university guzheng education may be seen as one of the settings where the concept of cultural sustainability is reflected in the learning experience of students. It includes both physical practice of performance, aesthetic sense, historical awareness, cultural identity, creative action, and social communication. Those characteristics of guzheng education provide it with appropriate conditions to develop and test a measure of students perceived cultural sustainability competence in a particular domain.

### 2.2 Guzheng education as a context for cultural sustainability competence

Guzheng has evolved out of an older context of traditional performance into conservatory-based and university-based teaching. The new development of the guzheng includes changes to performance practice, repertoire construction, professional training, stage presentation, and cultural identity (Sun, 2015; Silverberg, 2022; Han, 2013). As a traditional Chinese musical instrument, the guzheng is more than just an object that produces sounds or a means of performing. It contains aesthetic concepts, regional aesthetics, historical memory, embodied techniques, and symbolic meanings that are related to the musical culture of China.

Guzheng training in higher education typically involves technical practice, individual lessons, incremental learning of the repertoire, solo and ensemble playing and stage performance. The given system helped the professionalization and modern development of guzheng performance (Sun, 2015; Silverberg, 2022). Nevertheless, if university guzheng instruction is seen only in terms of technical proficiency, the competitive preparedness or the completeness of the repertoire, its more extensive cultural function may be neglected. A cultural sustainability standpoint would ask what understanding students have of the cultural worth of the instrument, what meanings they give to either traditional or contemporary repertoire, and what view they have of their future as performers, teachers, cultural facilitators, and transmitters of heritage.

The study of music education has long maintained that musical learning is a cultural practice of social significance (Lau, 2008; Schippers, 2010; Elliott and Silverman, 2015). This means that the musical competence of students cannot be separated into identity, participation, community and cultural understanding. As recent research on Chinese music education reveals, there is increasing awareness of cultural responsiveness, ethnic identity, multicultural education, and sustainability of traditional music education (Du and Leung, 2022; Guan et al., 2023; Yang and Welch, 2023). The implication of these studies is that traditional music education must not be restricted to the reproduction of individual technical formats. It is also necessary to encourage students to think critically and creatively about musical heritage.

This topic is particularly relevant to guzheng education since the instrument can be found in various places: universities and conservatories, concert halls, school classrooms, community cultural activities, online sites and events, and mass cultural activities. The creation of these spaces offers new opportunities in cultural communication, but it also demands more extensive competencies of the students. Students must be able to describe guzheng culture to non-specialist groups, be capable of differentiating between meaningful innovation and superficial commercialization, be responsible in their use of digital media, and relate the preparation of performances to future professional and social careers. Due to this, the concept of guzheng cultural sustainability competence needs to be viewed as an object of multidimensionality, and not merely as a one dimensional indicator of performance success.

### 2.3 Digital transmission, innovation, and community engagement

The field of digital technology has become a critical component of the transmission of intangible cultural heritage. The online platforms, digital documentation, interactive design, and virtual dissemination may help in making cultural traditions more available to more people (Yu, 2023; Cai et al., 2024). The Chinese traditional instrumental culture has also studied digital and interactive methods such as research on the sound design of guqin and interactive art (Shen et al., 2022). In the context of guzheng education, digital media can increase the range of learning materials, enhance their popularity, facilitate remote learning, and establish novel means of cultural communication.

Equally, the concept of digital transmission cannot be considered culturally sustainable by default. Short videos, online performances, and algorithm-based content can make things more visible but less culturally rich. The traditional music can also be modified into an entertainment item, a visual experience, or a marketable act. Thus, digital literacy in guzheng training must not only consist of using platforms. They must also be able to convey cultural values, vary their explanations to specific audiences, and maintain aesthetic and historical significance. Studies of guzheng education and distance learning have also indicated that digital devices could be used to develop professional and intercultural competencies only when they are integrated into well-considered pedagogical design (Yin, 2023).

Creativity is also an essential element. The modern guzheng performance has been further developed by new compositions, inter-genre collaboration, interdisciplinary performances, and media-based presentations (Sun, 2015; Silverberg, 2022). Such changes may assist conventional music to access a wider audience, though they also cause conflicts between tradition and innovation. Culturally speaking, innovation cannot be seen as an unrestrained novelty. It needs to be based on understanding traditional aesthetics, repertoire history, performance style and cultural meaning. A culturally competent guzheng student must be able to facilitate responsible innovation and identify the dangers of superficial or decontextualized adaptation.

The role of community engagement in the survival of traditional music is also essential. Intangible heritage relies on living practice, social participation, and intergenerational transmission (UNESCO, 2003; Smith and Akagawa, 2009). Community engagement in university guzheng education could cover school outreach, public lectures, community concerts, heritage education programs, cultural service activities, and cooperation with local cultural institutions. Through these activities students can go beyond test-based or competitive learning and relate the art of guzheng performance with cultural life in society. They also assist students in preparing their careers since they expand the potential connections between performance, teaching, cultural communication, and social service.

### 2.4 Proposed dimensions of guzheng cultural sustainability competence

The five proposed dimensions were derived through a synthesis of several overlapping theoretical and educational domains rather than from a single pre-existing competence framework. First, sustainability competence frameworks in higher education emphasize that competence extends beyond disciplinary knowledge to include values, judgment, participation, strategic action, communication, and the capacity to apply learning in social contexts (Wiek et al., 2011; Rieckmann, 2012; Brundiers et al., 2021; Lozano et al., 2017; Finnveden and Schneider, 2023). Second, cultural sustainability and intangible cultural heritage perspectives place particular emphasis on cultural continuity, identity, interpretation, living practice, community participation, and intergenerational transmission (UNESCO, 2003, 2019; Hawkes, 2001; United Cities and Local Governments, 2010; Soini and Birkeland, 2014; Dessein et al., 2015; Smith and Akagawa, 2009). Third, music education research conceptualizes musical learning as an embodied and culturally situated practice in which technical performance, aesthetic judgment, identity, interpretation, and participation are interconnected (Lau, 2008; Schippers, 2010; Elliott and Silverman, 2015; Du and Leung, 2022; Guan et al., 2023; Yang and Welch, 2023; Huang et al., 2025). Finally, research on digital heritage and technology-supported traditional music education highlights the growing importance of digital communication, audience engagement, and culturally responsible forms of online transmission (Yu, 2023; Cai et al., 2024; Shen et al., 2022; Yin, 2023). The proposed GCSCS dimensions were developed at the intersection of these four bodies of literature.

In the present study, student-perceived cultural sustainability competence is conceptualized as a multidimensional self-report construct reflecting students' perceived understanding, capability, judgment, and readiness to engage in culturally sustainable guzheng practice. This construct should be distinguished from objectively demonstrated competence and from narrower psychological constructs such as task-specific self-efficacy. Attitudes, beliefs, willingness, interests, and perceived ability are also not treated as interchangeable. Items referring to willingness, responsibility, interest, or belief are interpreted as dispositional or readiness-related indicators relevant to cultural participation and transmission, whereas ability-oriented items more directly reflect students' perceptions of what they can understand, explain, interpret, communicate, or apply. The GCSCS therefore represents a preliminary multidimensional self-report framework rather than a pure performance-based or ability-only measure of competence.

The first dimension, heritage identity and cultural responsibility, represents the cultural-continuity component of the framework. Cultural sustainability and intangible cultural heritage perspectives emphasize that heritage persists through recognition, interpretation, participation, and transmission rather than through preservation of objects alone (UNESCO, 2003, 2019; Hawkes, 2001; United Cities and Local Governments, 2010; Soini and Birkeland, 2014; Dessein et al., 2015; Smith and Akagawa, 2009). In guzheng education, this dimension therefore concerns students' understanding of the guzheng as cultural heritage, their identification with traditional musical culture, and their perceived responsibility for communicating and transmitting its cultural meanings. Research on culturally responsive and heritage-oriented music education further supports the relevance of identity and cultural understanding to traditional music learning (Guan et al., 2023; Yang and Welch, 2023; Huang et al., 2025).

The second dimension, embodied performance and aesthetic literacy, represents the enacted and interpretive component of cultural sustainability competence. Music education is not limited to declarative knowledge; musical understanding develops through bodily practice, listening, stylistic awareness, aesthetic judgment, and culturally informed interpretation (Lau, 2008; Schippers, 2010; Elliott and Silverman, 2015). For a performance tradition such as the guzheng, cultural continuity depends partly on whether students can connect technique and sound production with repertoire, style, aesthetic meaning, and historical context. This dimension therefore links domain-specific performance learning with the embodied transmission of musical culture.

The third dimension, creative adaptation and repertoire innovation, reflects the need to negotiate continuity and change. Broader sustainability competence frameworks emphasize judgment, value-sensitive decision making, and the capacity to act responsibly in changing contexts (Wiek et al., 2011; Rieckmann, 2012; Brundiers et al., 2021; Lozano et al., 2017; Finnveden and Schneider, 2023). Within contemporary guzheng practice, new compositions, cross-genre performance, interdisciplinary collaboration, and changing audience expectations create opportunities for innovation while also raising questions about cultural integrity and decontextualization (Sun, 2015; Silverberg, 2022; Han, 2013). This dimension therefore concerns students' judgment and readiness to engage in innovation while recognizing the aesthetic and cultural boundaries within which adaptation remains meaningful.

The fourth dimension, digital transmission and public communication, represents the outward communication component of the framework. Digital technologies have created new possibilities for documenting, teaching, presenting, and disseminating intangible cultural heritage (Yu, 2023; Cai et al., 2024; Shen et al., 2022). At the same time, increased visibility does not necessarily ensure culturally meaningful transmission. In guzheng education, students may need not only familiarity with digital platforms but also the capacity and readiness to explain repertoire, history, style, and aesthetic meaning to non-specialist audiences. This dimension therefore connects digital cultural participation with public-facing cultural interpretation and communication (Yu, 2023; Cai et al., 2024; Shen et al., 2022; Yin, 2023).

The fifth dimension, community engagement and sustainable career readiness, extends cultural sustainability competence from individual learning into social participation and longer-term professional practice. Sustainability competence frameworks emphasize interpersonal participation, collaboration, strategic action, and the application of learning beyond the classroom (Wiek et al., 2011; Rieckmann, 2012; Brundiers et al., 2021; Lozano et al., 2017; Finnveden and Schneider, 2023), while intangible cultural heritage depends on continuing community practice and social transmission (UNESCO, 2003; Smith and Akagawa, 2009). In guzheng education, these principles are reflected in school outreach, community education, heritage activities, cultural services, teaching, public communication, and professional pathways that connect artistic expertise with wider social and cultural roles.

Taken together, the five dimensions represent complementary functions required for culturally sustainable practice in this specific educational domain: heritage identity and cultural responsibility address cultural continuity; embodied performance and aesthetic literacy address embodied enactment and interpretation; creative adaptation and repertoire innovation address responsible change; digital transmission and public communication address outward cultural communication; and community engagement and sustainable career readiness address social participation and longer-term application. The five-dimensional model is therefore not intended as a direct replication of any single general sustainability competence framework. Rather, it is a domain-specific operationalization that integrates sustainability competence, cultural sustainability, intangible heritage transmission, music education, and contemporary forms of digital and community participation within university guzheng education. The present study does not assume that the same five-dimensional structure will necessarily apply unchanged to all areas of higher arts education; its broader transferability requires independent testing in other culturally embedded arts contexts.

### 2.5 Research model

The research model treats the five GCSCS dimensions as theoretically related but distinguishable domains of student-perceived cultural sustainability competence and readiness. Their expected relationships arise from a shared orientation toward culturally sustainable learning, but the dimensions represent different functions within that broader process. Heritage identity and embodied performance primarily concern students' cultural grounding and enacted understanding; creative adaptation and digital communication concern how that grounding is negotiated and communicated in changing contexts; and community engagement and career readiness extend learning into social participation and longer-term professional application.

The model therefore does not assume that strength in one dimension necessarily implies strength in another. A student may, for example, report strong heritage identity and embodied performance literacy while showing lower readiness for digital communication or community participation. Conversely, a student may report greater readiness for contemporary adaptation and digital communication without reporting equally strong traditional or aesthetic grounding. Treating the dimensions as related but non-redundant allows the GCSCS to examine these differentiated patterns rather than reducing cultural sustainability-related self-perceptions to a single undifferentiated outcome.

Accordingly, the five-dimensional structure was specified theoretically before the empirical factor analysis. The exploratory factor analysis was used to examine whether the theory-informed dimensional organization was empirically interpretable in the present sample, rather than to generate the conceptual dimensions solely from the observed data. This distinction is important because the GCSCS was developed as a theory-guided, domain-specific instrument whose proposed structure required empirical examination and subsequent refinement.

## 3 Materials and methods

### 3.1 Research design

The research design was a cross-sectional survey that developed and initially validated the Guzheng Cultural Sustainability Competence Scale (GCSCS). The study was an exploratory because there was no existing measure to use in measuring perceived cultural sustainability competence among university guzheng students. It was not to assess any particular teaching intervention or to offer a national representation of all of the guzheng students across China. Rather, the aim was to offer preliminary psychometric support to a context-specific instrument that can be applied to higher arts education.

The GCSCS was designed as a multidimensional self-report measure of students' perceived cultural sustainability competence in guzheng education. For the purposes of the present exploratory study, this construct was operationalized through four related forms of self-perception: perceived understanding of guzheng as cultural heritage, perceived capability to connect performance with aesthetic and cultural interpretation, judgment regarding responsible creative adaptation, and readiness for digital, public, community, and professional participation. These components were treated as theoretically related but not psychologically identical. In particular, willingness, beliefs, interests, and responsibility orientations were not regarded as direct equivalents of perceived ability. Accordingly, GCSCS scores should be interpreted as multidimensional student self-perceptions related to culturally sustainable practice rather than as objective evidence of demonstrated competence or as a pure measure of self-efficacy.

The research process included conceptual development, item generation, expert appraisal, questionnaire administration, data screening, item-level analysis, exploratory factor analysis, reliability analysis, descriptive analysis, correlation analysis, and cluster analysis. The conceptual framework was informed by literature on Education for Sustainable Development, sustainability competence, cultural sustainability, intangible cultural heritage, traditional music education, and guzheng education (UNESCO, 2017, 2020, 2024; Wiek et al., 2011; Lozano et al., 2017; Finnveden and Schneider, 2023; UNESCO, 2003; Sun, 2015; Silverberg, 2022; Han, 2013; Lau, 2008; Guan et al., 2023; Yang and Welch, 2023; Huang et al., 2025; Yin, 2023). Methodological guidance on scale development, exploratory factor analysis, reliability testing, and cluster analysis was also considered during the design and analysis of the study (Boateng et al., 2018; DeVellis and Thorpe, 2021; Costello and Osborne, 2005; Hair et al., 2019; Kaufman and Rousseeuw, 1990).

### 3.2 Participants and sampling

Participants were recruited from five higher education institutions in Northeast China. The participating institutions included a comprehensive university, conservatories, and teacher education universities with music or arts programs. To protect institutional and participant confidentiality, the names of the institutions were not reported in the manuscript. They were coded as Institution A–E (Table 1).

**Table 1:** Institutional sampling structure.

Institution code	Institution type	Regional scope	Target participants
Institution A	Comprehensive university	Northeast China	Guzheng students
Institution B	Conservatory	Northeast China	Guzheng students
Institution C	Teacher education university	Northeast China	Guzheng students
Institution D	Teacher education university	Northeast China	Guzheng students
Institution E	Conservatory	Northeast China	Guzheng students

The participating institutions are anonymized as Institution A–E to protect institutional and participant confidentiality. The study used a multi-institutional regional sample rather than a nationally representative sample.

The final sample included 168 university students majoring in guzheng performance or related guzheng education programs. Most participants were undergraduate students, while a smaller number were postgraduate students. The sampling strategy was intended to include students from different types of higher arts education environments within a regional context. It was not designed to produce a nationally representative sample. Therefore, the findings should be interpreted as preliminary evidence from a multi-institutional regional sample.

### 3.3 Instrument development and expert appraisal

The initial GCSCS was developed to measure students' perceived cultural sustainability competence in university guzheng education. Item development followed two principles. First, each item had to correspond to a theoretically meaningful dimension of guzheng-related cultural sustainability competence. Second, the wording had to be understandable to university guzheng students and closely related to their learning experience.

The initial scale contained 20 items across five proposed dimensions, with four items for each dimension. The five dimensions were heritage identity and cultural responsibility, embodied performance and aesthetic literacy, creative adaptation and repertoire innovation, digital transmission and public communication, and community engagement and sustainable career readiness. All items were written as self-report statements and measured using a five-point Likert scale, where 1 = strongly disagree and 5 = strongly agree.

The five-dimensional structure was specified *a priori* on the basis of the theoretical synthesis presented in Section 2.4. The dimensions were therefore not generated retrospectively from the factor-analytic results. Rather, exploratory factor analysis was subsequently used to examine the extent to which the theoretically proposed organization was supported, modified, or challenged by the observed item-loading patterns.

The first aspect, which is heritage identity and cultural responsibility, measured whether students perceived guzheng learning as a mode of cultural heritage transmission or not only as technical training. The second aspect, embodied performance and aesthetic literacy measured the ability of students to perceive techniques, tone production, style, listening and cultural inference. The third aspect, creative adaptation and repertoire innovation measured the perspective of students on responsible innovation and current adaptation. The fourth aspect, digital transmission and public communication measured the willingness of the students and their perceived ability to communicate the guzheng culture by using digital media and public communication. The fifth aspect, community involvement and sustainability-oriented career preparedness measured the perception of students on the relationship between learning guzheng, cultural outreach, education to the community, and future career possibilities.

The initial item pool contained both capability-oriented and disposition- or readiness-oriented statements. Capability-oriented items referred more directly to what students perceived that they could explain, interpret, distinguish, communicate, or integrate in guzheng-related practice. Other items referred to understanding, responsibility, willingness, beliefs, or interest in culturally sustainable participation. In the revised conceptualization, these latter items are not treated as direct measures of perceived ability. Instead, they are regarded as indicators of students' perceived orientation or readiness toward cultural participation, transmission, and professional engagement. This distinction is important because the GCSCS was developed as a broad, domain-specific self-report instrument and not as an ability-only or performance-based assessment.

The survey was carried out in Chinese. The questionnaire was administered in Chinese. The English version reported in this manuscript was prepared for academic reporting and closely follows the content of the Chinese questionnaire used in data collection. The wording of the items were made as similar as possible to the initial survey questions to make the English reporting version resemble the instrument that has been used in the process of collecting data.

To strengthen content validity evidence, an expert appraisal was conducted for the initial 20-item pool. The expert panel consisted of five specialists with backgrounds in guzheng performance, guzheng pedagogy, music education, educational measurement, and traditional Chinese music culture. Experts evaluated each item in terms of relevance, clarity, dimensional fit, and cultural appropriateness. Their ratings were used to examine whether the initial item pool adequately represented the proposed five-dimensional construct. The expert panel was anonymized in the manuscript; only their areas of expertise were reported (Table 2).

**Table 2:** Theoretical basis and initial dimensions of the Guzheng Cultural Sustainability Competence Scale.

Dimension	Code	Primary theoretical linkage	Domain-specific operational emphasis	Sample item
Heritage identity and cultural responsibility	HI	Cultural sustainability and intangible cultural heritage (UNESCO, 2003, 2019; Hawkes, 2001; United Cities and Local Governments, 2010; Soini and Birkeland, 2014; Dessein et al., 2015; Smith and Akagawa, 2009)	Heritage understanding, cultural identity, and responsibility for transmission	I can explain the cultural meaning of selected guzheng works to audiences or learners.
Embodied performance and aesthetic literacy	EP	Embodied and culturally situated music education (Lau, 2008; Schippers, 2010; Elliott and Silverman, 2015)	Integration of technique, sound, style, aesthetic judgment, and cultural interpretation	I can connect finger technique, tone production, and musical expression in guzheng performance.
Creative adaptation and repertoire innovation	CA	Sustainability competence and responsible adaptation in contemporary guzheng practice (Wiek et al., 2011; Rieckmann, 2012; Brundiers et al., 2021; Lozano et al., 2017; Finnveden and Schneider, 2023; Sun, 2015; Silverberg, 2022; Han, 2013)	Judgment regarding culturally meaningful innovation and adaptation	I can distinguish meaningful innovation from superficial commercialization in guzheng performance.
Digital transmission and public communication	DT	Digital cultural heritage and technology-supported music education (Yu, 2023; Cai et al., 2024; Shen et al., 2022; Yin, 2023)	Digital/public communication of guzheng knowledge while retaining cultural context	I can present guzheng-related knowledge in a way that is understandable to non-specialist audiences.
Community engagement and sustainable career readiness	CE	Participation-oriented sustainability competence and living heritage transmission (Wiek et al., 2011; Rieckmann, 2012; Brundiers et al., 2021; Lozano et al., 2017; Finnveden and Schneider, 2023; UNESCO, 2003; Smith and Akagawa, 2009)	Community participation, cultural outreach, social service, and professional pathways	I can see possible career pathways that combine guzheng performance, teaching, cultural communication, and social engagement.

The theoretical linkages indicate the primary literature domains informing each proposed dimension; the dimensions were derived through synthesis rather than through one-to-one adoption of an existing framework. The sample items are drawn from the initial questionnaire administered during data collection. The complete instrument is provided in Appendix A.

### 3.4 Data collection procedure

Data were collected through an anonymous questionnaire survey. The questionnaire consisted of two parts. The first part collected background information, including gender, age, educational level, year of study, years of guzheng learning, weekly practice time, competition experience, public performance experience, community or heritage-related activity experience, and digital dissemination experience. The second part contained the initial 20 GCSCS items.

Participation was voluntary. Before completing the questionnaire, participants were informed of the purpose of the study, the anonymous nature of the survey, and their right to withdraw before submission. No personally identifiable information, such as names, student numbers, class identifiers, or institutional names, was included in the dataset used for analysis. The questionnaire was distributed to eligible guzheng students through institutional or course-related communication channels. Only completed questionnaires from eligible guzheng students were included in the final analysis.

The questionnaire was brief in order to limit the response load. The questions were phrased in such a way that they would capture the routine learning experience of students and their professional training environment. Five point Likert format was chosen as it enabled the respondents to indicate various levels of agreement without changing the ease of responding.

### 3.5 Data analysis

Data analysis was conducted in several steps. First, the dataset was screened for completeness, coding accuracy, duplicate responses, missing values, and out-of-range values. Institutional identifiers were anonymized as Institution A–E before analysis. Variables used only for data organization or draft-stage analysis were not included in the formal analyses (Table 3).

**Table 3:** Overview of data analysis procedures.

Step	Analysis	Purpose
1	Data screening	To check completeness, coding accuracy, duplicate responses, missing values, out-of-range values, and exclusion of non-formal auxiliary fields
2	Expert appraisal	To evaluate item relevance, clarity, dimensional fit, and cultural appropriateness
3	Item screening	To examine item means, standard deviations, corrected item-total correlations, and reliability if item deleted
4	Exploratory factor analysis	To examine the empirical interpretability of the theory-specified five-dimensional structure using principal axis factoring with oblimin rotation and inspection of primary and secondary loading patterns
5	Reliability analysis	To assess internal consistency of the subscales and full scale using Cronbach's α and McDonald's ω
6	Descriptive statistics	To summarize participant characteristics and dimension scores
7	Correlation analysis	To examine associations among the retained dimensions
8	To explore learner-profile patterns using z-standardized subscale scores and compare three-, four-, and five-cluster solutions using diagnostic and substantive criteria	To identify differentiated learner profiles based on standardized subscale scores

Cluster analysis was based on standardized subscale scores generated from the retained GCSCS dimensions. Student profiles were treated as analytical results rather than original questionnaire variables.

Second, expert appraisal results were summarized to provide content validity evidence for the initial item pool. Item-level content validity index values and scale-level average content validity index values were calculated for item relevance, clarity, dimensional fit, and cultural appropriateness. These results were used to support the theoretical coverage of the initial 20-item GCSCS.

Third, item-level screening was conducted before factor interpretation. Item means, standard deviations, corrected item-total correlations, and Cronbach's α if item deleted were examined for the initial 20 items. Item screening was used to identify items that required closer inspection, but it was not used as the sole basis for item deletion. Final item retention was determined by considering item-level statistics, factor loading patterns, cross-loading patterns, and theoretical interpretability together.

Fourth, Exploratory Factor Analysis (EFA) was conducted to examine the empirical organization of the proposed GCSCS dimensions. The suitability of the data for factor analysis was evaluated using the Kaiser–Meyer–Olkin (KMO) measure and Bartlett's test of sphericity. Principal axis factoring was used as the extraction method, and direct oblimin rotation was applied because the proposed dimensions were expected to be correlated. Because the five-dimensional structure had been specified theoretically before the empirical analysis, a five-factor solution was examined as a theory-guided exploratory model rather than treating factor retention as a purely data-driven procedure. The purpose of the EFA was therefore to evaluate whether the item-response patterns were interpretable in relation to the proposed dimensions and to identify items whose empirical placement challenged the theoretical structure.

For reporting and interpretation, primary loadings of approximately 0.40 or higher were treated as minimally acceptable in this preliminary scale-development context. Secondary loadings around 0.30 or higher were flagged for closer examination rather than used as an automatic deletion criterion. Item-retention decisions were based on the combined consideration of primary loading strength, cross-loading patterns, communality, corrected item–total relationships, substantive item meaning, and theoretical alignment. An item was therefore not removed solely because it showed a secondary loading, nor was a satisfactory item–total correlation considered sufficient evidence for dimensional retention. The initial 20-item solution was examined first, followed by a revised solution after removal of items whose loading patterns did not correspond clearly to their theoretically assigned dimensions.

Fifth, internal consistency reliability was assessed using Cronbach's α and McDonald's ω for each retained subscale and for the full scale. Values around or above 0.70 were considered acceptable for preliminary scale development. Reliability results were interpreted together with the factor structure and theoretical coherence of the retained items rather than as isolated indicators.

Sixth, descriptive statistics were calculated to summarize participant characteristics and dimension scores. Means and standard deviations were used to describe the five retained competence dimensions and to identify relative strengths and weaker areas in students' perceived guzheng cultural sustainability competence.

Seventh, Pearson correlation analysis was conducted to examine relationships among the five retained dimensions. This analysis was used to determine whether the dimensions were positively related but not redundant.

Finally, an exploratory k-means cluster analysis was conducted using the five retained GCSCS subscale scores. The subscale scores were z-standardized before clustering so that differences in scale dispersion would not disproportionately influence the solution. The purpose of the analysis was descriptive rather than confirmatory: it was used to examine whether students showed recurring multivariate score patterns across the five dimensions.

Three-, four-, and five-cluster solutions were compared. Solution selection was based on multiple considerations rather than a single fit index, including cluster size, silhouette coefficient, within-cluster dispersion, centroid separation, parsimony, and substantive interpretability of the resulting score patterns. The four-cluster solution was retained because it produced four interpretable profiles with acceptable group sizes while preserving distinctions that were obscured in the three-cluster solution. Importantly, the four-cluster solution did not maximize the silhouette coefficient. Accordingly, the selected solution was treated as an exploratory descriptive representation rather than evidence of four naturally occurring or definitive student types. Cluster labels were assigned after inspection of the dimension-score patterns and were not predetermined categories. Full comparisons of the three-, four-, and five-cluster solutions are reported in Supplementary Table S4.

Primary analyses, including descriptive statistics, item screening, exploratory factor analysis, reliability analysis, correlation analysis, and k-means clustering, were conducted using IBM SPSS Statistics 26.0. McDonald's ω and cluster diagnostic indices were calculated from the same anonymized dataset using supplementary statistical software.

### 3.6 Ethical considerations

The study was conducted in accordance with ethical principles for research involving human participants. Ethical review and approval were not required because the study was an anonymous, non-experimental educational survey involving minimal risk, no intervention, and no collection of personally identifiable information. Participation was voluntary. Before completing the questionnaire, participants were informed of the purpose of the study, the anonymous nature of the survey, the intended use of aggregated data for academic publication, and their right to withdraw before submission. Submission of the completed questionnaire was regarded as informed consent. The dataset used for analysis did not include names, student numbers, class identifiers, or institutional names. The participating institutions were anonymized as Institution A–E, and all findings were reported only in aggregate form.

## 4 Results

### 4.1 Content validity evidence and participant characteristics

The expert appraisal provided initial content validity evidence for the 20-item GCSCS. All 20 items were accepted for initial field testing. The overall S-CVI/Ave was 0.9775. The criterion-specific S-CVI/Ave values were 1.00 for item relevance, 0.96 for clarity, 0.98 for dimensional fit, and 0.97 for cultural appropriateness. The minimum average item CVI was 0.85. Two items, CA4 and DT4, were noted as requiring attention because they involved possible construct-boundary issues between creative adaptation and digital communication. However, the expert appraisal did not predetermine item retention; the final decision was based on the exploratory factor analysis and theoretical interpretability.

The formal analysis considered all 168 valid responses. The participants were all guzheng students of five higher education institutions in Northeast China. The majority of the sample were females. Most of the participants were undergraduate students and fewer of them were postgraduate students. On average, the students had been learning guzheng for 9.49 years and practiced 10.54 h per week.

Regarding learning and experience in performance, 66.7 percent of the students mentioned that they have experienced competition and 80.4 percent of them mentioned that they had experienced a public performance. In contrast, only 42.9 percent had engaged in community or heritage-oriented guzheng events, and 56.0 percent had experience posting guzheng performances or related information online. This trend indicates that experience-oriented performance was a typical characteristic of the sample, but experience in the community and digital media-based cultural participation were not as usual (Table 4).

**Table 4:** Participant characteristics.

Variable	Category/Indicator	*n*/Mean	%/SD
Total valid responses	—	168	100.0
Gender	Female	139	82.7
	Male	29	17.3
Education level	Undergraduate students	151	89.9
	Postgraduate students	17	10.1
Year of study	Year 1	38	22.6
	Year 2	44	26.2
	Year 3	41	24.4
	Year 4	28	16.7
	Postgraduate	17	10.1
Age	Mean ± SD	20.73	1.64
Guzheng learning experience	Years of guzheng learning	9.49	2.59
Practice time	Weekly practice time, hours	10.54	3.54
Competition experience	Yes	112	66.7
	No	56	33.3
Public performance experience	Yes	135	80.4
	No	33	19.6
Community or heritage-related activity experience	Yes	72	42.9
	No	96	57.1
Digital dissemination experience	Yes	94	56.0
	No	74	44.0

The participating institutions were anonymized as Institution A–E. Percentages are based on the total valid sample, *N* = 168. For continuous variables, the last column reports standard deviations.

### 4.2 Item screening

Item-level screening was conducted for the initial 20 GCSCS items before the factor structure was interpreted. Item means ranged from 3.33 to 4.33, and standard deviations ranged from 0.62 to 1.00. Corrected item-total correlations ranged from 0.405 to 0.663. These values indicated that all items had acceptable item-total relationships at the preliminary screening stage.

CA4 and DT4 did not show poor corrected item-total correlations. Their corrected item-total correlations were 0.509 and 0.575, respectively. Therefore, they were not removed on the basis of item-total statistics. Instead, both items were retained for exploratory factor analysis and were later evaluated according to their factor loading patterns and theoretical alignment. This distinction is important because corrected item-total correlations indicate the extent to which an item is associated with the overall scale, but they do not show whether the item belongs clearly to its intended dimension.

### 4.3 Exploratory factor analysis

Exploratory factor analysis was first conducted on the initial 20-item GCSCS. The data were suitable for factor analysis, with KMO = 0.895 and Bartlett's test of sphericity significant, χ^2^(190) = 1,411.905, *p* < 0.001. The five extracted factors explained 52.46% of the total variance in the initial 20-item solution. Consistent with the theory-guided analytic strategy described in Section 3.5, the five-factor solution was examined because the instrument had been developed around five theoretically specified dimensions. The EFA was used to evaluate the empirical coherence of this proposed organization rather than to claim that five factors constituted the only possible data-driven solution.

Two items showed particularly unclear dimensional alignment in the initial solution. CA4, which had been theoretically assigned to creative adaptation and repertoire innovation, loaded only weakly on its intended factor (0.129) and most strongly on digital transmission and public communication (0.461). DT4, theoretically assigned to digital transmission and public communication, loaded more strongly on creative adaptation (0.432) than on its intended digital factor (0.348). Their corrected item–total correlations were not problematic; rather, the concern was dimensional placement. Taken together with the substantive content of the two items, these loading patterns suggested that CA4 and DT4 reflected a boundary between contemporary creative production and digital practice rather than clear indicators of their originally assigned dimensions. The two items were therefore removed before the final 18-item solution was examined.

After CA4 and DT4 were removed, the final 18-item solution was examined. The final dataset remained suitable for factor analysis, with KMO = 0.882. Bartlett's test of sphericity was significant, χ^2^(153) = 1,228.887, *p* < 0.001. The five extracted factors explained 53.35% of the total variance. The retained factors were conceptually interpretable as heritage identity and cultural responsibility, embodied performance and aesthetic literacy, creative adaptation and repertoire innovation, digital transmission and public communication, and community engagement and sustainable career readiness.

EP2 warrants particular attention because its loading pattern was less distinct than those of most other retained items. In the initial 20-item solution, EP2 loaded 0.418 on its intended embodied performance and aesthetic literacy factor and showed a secondary loading of 0.355 on heritage identity and cultural responsibility, with a communality of 0.513. In the final 18-item solution, its primary loading on the intended factor was 0.411. We retained EP2 because its highest loading remained on the theoretically intended factor, its primary loading remained above the minimum interpretive threshold used in this preliminary EFA, its communality and corrected item–total relationship were acceptable, and its substantive content remained relevant to stylistic and contextual awareness within embodied musical learning. Nevertheless, the relatively close secondary loading indicates that EP2 may partly bridge embodied aesthetic literacy and heritage understanding. Its dimensional specificity should therefore be re-examined in an independent confirmatory study. The factor loadings, communalities, and corresponding interpretations for the final 18-item GCSCS are presented in Table 5.

**Table 5:** Exploratory factor analysis results for the final 18-item GCSCS.

Factor	Item	Primary loading	Communality	Interpretation
Factor 1	HI1	0.705	0.521	Heritage identity and cultural responsibility
	HI2	0.579	0.393	
	HI3	0.545	0.506	
	HI4	0.601	0.434	
Factor 2	EP1	0.605	0.476	Embodied performance and aesthetic literacy
	EP2	0.411	0.511	
	EP3	0.531	0.419	
	EP4	0.751	0.592	
Factor 3	CA1	0.779	0.642	Creative adaptation and repertoire innovation
	CA2	0.511	0.414	
	CA3	0.675	0.523	
Factor 4	DT1	0.854	0.721	Digital transmission and public communication
	DT2	0.763	0.585	
	DT3	0.761	0.689	
Factor 5	CE1	0.671	0.490	Community engagement and sustainable career readiness
	CE2	0.734	0.605	
	CE3	0.548	0.480	
	CE4	0.607	0.601	

Principal axis factoring with direct oblimin rotation was used. The five-factor solution was examined as a theory-guided exploratory structure based on the dimensions specified before empirical analysis. Only primary loadings are shown in the main table for readability; the complete pattern matrix for the initial 20-item solution, including secondary loadings, is provided in Supplementary Table S3. Item retention was based on loading patterns, communality, item-level statistics, substantive meaning, and theoretical alignment rather than on a single mechanical cutoff.

### 4.4 Reliability and descriptive statistics

Internal consistency was assessed for the final 18-item GCSCS. The full scale showed good reliability, with Cronbach's α = 0.893 and McDonald's ω = 0.897. The five subscales also showed acceptable to good internal consistency. Cronbach's α ranged from 0.752 to 0.846, and McDonald's ω ranged from 0.757 to 0.846 (Table 6).

**Table 6:** Descriptive statistics and reliability of the final GCSCS.

Dimension	Items retained	Mean	SD	Cronbach's α	McDonald's ω
Heritage identity and cultural responsibility	4	4.24	0.50	0.766	0.766
Embodied performance and aesthetic literacy	4	4.12	0.52	0.783	0.784
Creative adaptation and repertoire innovation	3	3.70	0.67	0.752	0.757
Digital transmission and public communication	3	3.51	0.85	0.846	0.846
Community engagement and sustainable career readiness	4	3.60	0.69	0.816	0.817
Full scale	18	3.83	0.48	0.893	0.897

Scores were measured on a five-point Likert scale from 1, strongly disagree to 5, strongly agree. Means are presented descriptively to compare relative score patterns within the present sample; no validated cut-off values were used to classify students or subscales into proficiency levels.

The descriptive results showed a clear pattern across the five dimensions. Students reported the highest scores for heritage identity and cultural responsibility (*M* = 4.24, SD = 0.50) and embodied performance and aesthetic literacy (*M* = 4.12, SD = 0.52). Creative adaptation and repertoire innovation showed a moderately high mean score (*M* = 3.70, SD = 0.67). Digital transmission and public communication had the lowest mean score among the five dimensions (*M* = 3.51, SD = 0.85), and it also showed the largest standard deviation. Community engagement and sustainable career readiness remained at a moderate-to-moderately-high level (*M* = 3.60, SD = 0.69).

Descriptively, heritage identity and cultural responsibility and embodied performance and aesthetic literacy had the highest mean scores, whereas digital transmission and public communication and community engagement and sustainable career readiness had lower mean scores. These values describe relative score patterns within the present sample and are not intended as externally validated proficiency levels. The relative mean scores of the five GCSCS dimensions are illustrated in Figure 1.

**Figure 1:**
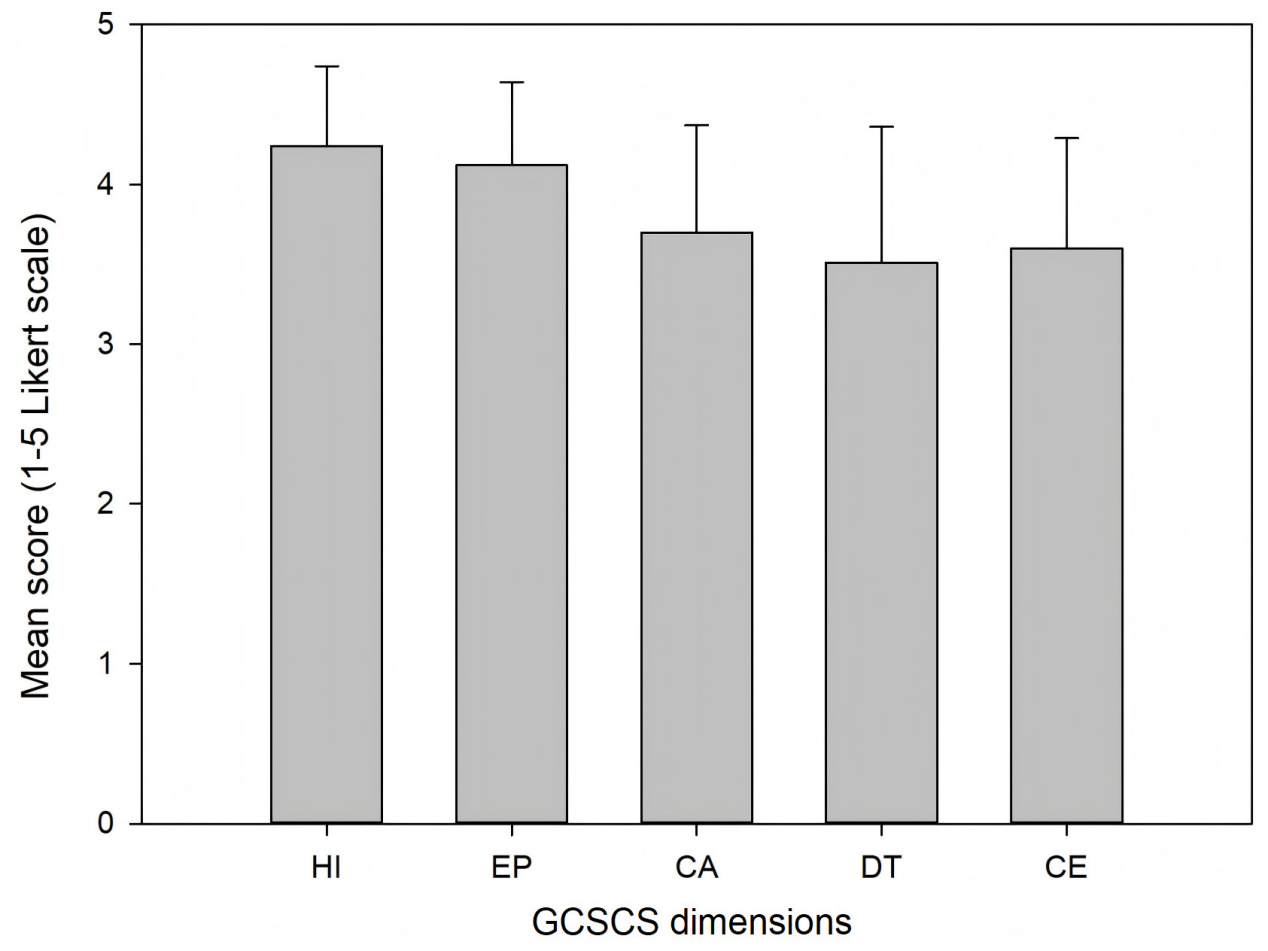
Mean scores of the five dimensions of Guzheng Cultural Sustainability Competence. Bar chart comparing the mean self-reported scores of 168 university guzheng students across five Guzheng Cultural Sustainability Competence Scale (GCSCS) dimensions. The horizontal axis lists heritage identity (HI), embodied performance literacy (EP), creative adaptation (CA), digital transmission (DT), and community engagement (CE). The vertical axis shows mean scores on a five-point Likert scale. HI has the highest mean (4.24), followed by EP (4.12), CA (3.70), CE (3.60), and DT (3.51). Vertical error bars show standard deviations. The pattern indicates relatively higher self-reported heritage and performance scores and lower digital and community-related scores within this sample.

### 4.5 Correlations among the five dimensions

Pearson correlation analysis was conducted to examine associations among the five retained dimensions. All correlations were positive. The coefficients ranged from 0.254 to 0.594, suggesting that the dimensions were related but not redundant (Table 7).

**Table 7:** Correlations among the five GCSCS dimensions.

Dimension	HI	EP	CA	DT	CE
HI	1.000	0.594	0.414	0.262	0.555
EP	0.594	1.000	0.390	0.254	0.514
CA	0.414	0.390	1.000	0.518	0.516
DT	0.262	0.254	0.518	1.000	0.407
CE	0.555	0.514	0.516	0.407	1.000

Pearson correlations were calculated from final dimension scores, *N* = 168. HI, heritage identity and cultural responsibility; EP, embodied performance and aesthetic literacy; CA, creative adaptation and repertoire innovation; DT, digital transmission and public communication; CE, community engagement and sustainable career readiness.

The strongest association was observed between heritage identity and embodied performance and aesthetic literacy (*r* = 0.594). Heritage identity was also moderately associated with community engagement and sustainable career readiness (*r* = 0.555). Creative adaptation was moderately associated with digital transmission (*r* = 0.518) and community engagement (*r* = 0.516). These patterns indicate that the five dimensions shared a common competence orientation while retaining distinguishable meanings.

### 4.6 Learner profiles based on cluster analysis

Cluster analysis was conducted using z-standardized scores for the five retained GCSCS dimensions. Three-, four-, and five-cluster solutions were compared. The three-cluster solution yielded cluster sizes of 42, 82, and 44 and a silhouette coefficient of 0.229. The four-cluster solution yielded cluster sizes of 31, 44, 48, and 45 and a silhouette coefficient of 0.223, whereas the five-cluster solution yielded cluster sizes of 37, 26, 33, 31, and 41 and a lower silhouette coefficient of 0.192. Thus, the four-cluster solution did not provide the highest silhouette value; the three-cluster solution was marginally higher on this index.

The four-cluster solution was nevertheless retained for exploratory description because it maintained acceptable group sizes and separated score patterns that were merged in the three-cluster solution, while the five-cluster solution provided lower silhouette separation and a less parsimonious subdivision of the sample. The relatively small difference between the three- and four-cluster silhouette coefficients also indicates that cluster separation was modest. The four-profile solution should therefore be interpreted as a descriptive and provisional representation of learner heterogeneity rather than as evidence of four definitive or naturally occurring student categories. Full diagnostic comparisons are provided in Supplementary Table S4.

The four profiles were labeled balanced cultural competence learners, tradition-oriented performers, innovation-ready communicators, and skill-focused learners with limited cultural engagement. These labels were assigned after examining the profile means across the five retained dimensions.

The first profile, balanced cultural competence learners, represented 28.6% of the sample. Students in this group scored high across all five dimensions. Their profile suggests a relatively integrated competence pattern, with strong heritage identity, embodied performance literacy, creative adaptation, digital communication readiness, and community engagement readiness.

The second profile, which is tradition-oriented performers, was 26.2 percent of the sample. The students in this group had high heritage identity and embodied performance literacy, low digital transmission and public communication. Such a trend indicates that there is a very solid traditional learning background with a less developed ability to communicate outwardly.

Innovation ready communicators made up the third profile and constituted 26.8 percent of the sample. The students in this category had relatively high scores on creative adaptation and digital transmission. This group was found to have lower levels of heritage identity and embodied performance literacy as compared to the tradition oriented performer group, indicating a profile that is more prepared to communicate in contemporary ways but is not grounded in tradition. The descriptive characteristics and mean scores of the four exploratory learner profiles are summarized in Table 8.

**Table 8:** Learner profiles based on cluster analysis.

Profile	*n*	%	HI	EP	CA	DT	CE
Balanced cultural competence learners	48	28.6	4.58	4.58	4.27	4.09	4.15
Tradition-oriented performers	44	26.2	4.52	4.33	3.45	3.03	3.76
Innovation-ready communicators	45	26.8	4.03	3.77	3.93	3.96	3.49
Skill-focused learners with limited cultural engagement	31	18.5	3.61	3.61	2.83	2.61	2.67

Cluster analysis was conducted using standardized subscale scores. The values reported in the table are mean scores for each profile on the original five-point scale. HI, heritage identity and cultural responsibility; EP, embodied performance and aesthetic literacy; CA, creative adaptation and repertoire innovation; DT, digital transmission and public communication; CE, community engagement and sustainable career readiness.

The fourth profile is skill-focused learners with low cultural engagement and constitutes 18.5 % of the sample. The participants in that category had the least scores on creative adaptation, digital transmission as well as community engagement and they also exhibited comparatively lesser levels of heritage identity and performance literacy. This profile indicates that there is a group of learners who might require additional systematic assistance to link technical training to cultural values, social interactions, and overall professional advancement. The relative score patterns of the four exploratory learner profiles across the five GCSCS dimensions are illustrated in Figure 2.

**Figure 2:**
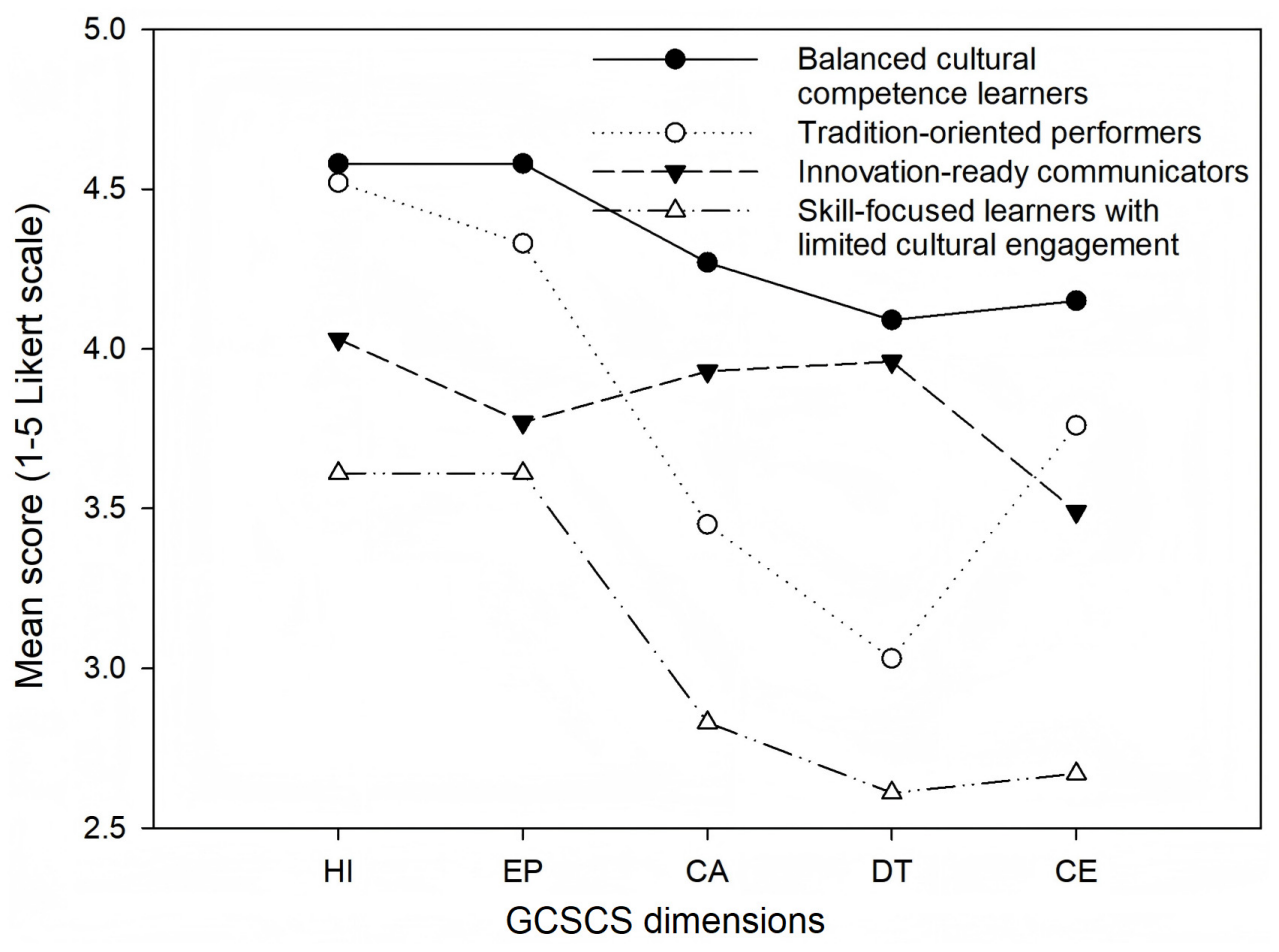
Learner profiles based on the five GCSCS dimensions. Line chart comparing four exploratory learner profiles across five GCSCS dimensions: heritage identity (HI), embodied performance literacy (EP), creative adaptation (CA), digital transmission (DT), and community engagement (CE). The horizontal axis lists the dimensions; the vertical axis shows mean scores on a five-point Likert scale. Balanced cultural competence learners show relatively high scores across all dimensions. Tradition-oriented performers score higher on HI and EP than on DT. Innovation-ready communicators show comparatively higher scores on CA and DT. Skill-focused learners with limited cultural engagement generally have the lowest scores across dimensions. The lines describe group-level self-reported score patterns, not fixed learner categories.

### 4.7 Summary of key findings

The analyses provide preliminary evidence regarding the organization and internal consistency of the GCSCS. Expert appraisal supported the initial content coverage, and item screening did not reveal serious item–total problems. In the EFA, CA4 and DT4 showed loading patterns that did not correspond clearly to their intended dimensions. After these two items were removed, the final 18-item solution showed an interpretable five-factor pattern and acceptable internal consistency across the retained subscales.

Descriptively, heritage identity and cultural responsibility and embodied performance and aesthetic literacy had higher mean scores than digital transmission and public communication and community engagement and sustainable career readiness in this sample. The five subscales were positively associated but not redundant. Exploratory cluster analysis yielded four descriptive score-pattern profiles, although the separation among candidate solutions was modest. These findings should therefore be understood as preliminary evidence about students' self-reported perceptions rather than as indicators of objectively demonstrated competence or stable learner types.

## 5 Discussion

### 5.1 Main findings

This study developed and preliminarily evaluated the GCSCS as a domain-specific self-report instrument for examining students' cultural sustainability-related perceptions in university guzheng education. The final 18-item version showed an interpretable five-factor structure and acceptable internal consistency across the five retained dimensions. These findings provide preliminary support for the proposed multidimensional organization but do not constitute full validation of the instrument.

The descriptive results showed unequal score patterns across the five dimensions. Heritage identity and cultural responsibility and embodied performance and aesthetic literacy received relatively higher ratings, whereas digital transmission and public communication and community engagement and sustainable career readiness received lower ratings. These differences describe how students in the present sample responded to the instrument; they do not by themselves establish differences in objectively demonstrated competence or demonstrate the effects of particular curricular practices.

The exploratory cluster analysis further indicated that different combinations of subscale scores could be represented within the sample. The selected four-cluster solution is useful as a descriptive way of illustrating learner heterogeneity, but the modest cluster separation means that the profiles should not be interpreted as fixed student types or as direct evidence that particular groups require specific interventions.

### 5.2 Construct scope and interpretation of the GCSCS

The GCSCS should be interpreted as a multidimensional self-report measure of culturally sustainable learning-related self-perceptions rather than as a direct assessment of objectively demonstrated competence. Its retained dimensions capture related aspects of perceived understanding, capability, judgment, and readiness in guzheng education. This distinction is important because individual items do not all represent the same psychological process. Some items directly ask students about perceived capability, whereas others concern responsibility, willingness, beliefs, or interest in culturally sustainable participation.

Accordingly, the term “competence” is used in this study at the level of a broader educational construct rather than to imply that every item constitutes a direct ability statement. Dispositional and readiness-related items may be relevant to participation in cultural transmission, digital communication, community engagement, and professional development, but they should not be interpreted as equivalent to perceived ability. The present findings therefore provide preliminary evidence for the organization of these domain-specific self-perceptions, while further research is needed to determine whether capability-related and dispositional components can be more clearly differentiated psychometrically.

The expert appraisal and item screening results offered initial support for the content coverage of the scale. The high S-CVI/Ave values suggest that the initial item pool was judged to be relevant and culturally appropriate by specialists. At the same time, the EFA results showed that expert judgement alone was not sufficient for final item retention. CA4 and DT4 were not removed because they were weak in general item-total relationships; rather, their empirical loading patterns did not align clearly with their intended theoretical dimensions. This strengthens the methodological logic of the study because item retention was based on both theoretical and empirical evidence.

### 5.3 Interpreting the five-dimensional structure

The five-dimensional structure should be interpreted as a domain-specific organization of related cultural sustainability functions rather than as five unrelated competencies. Heritage identity and embodied performance literacy represent cultural grounding and enacted understanding; creative adaptation represents the negotiation of continuity and change; digital transmission represents outward cultural communication; and community engagement and career readiness extend learning into social and professional participation. In this sense, the empirical structure broadly corresponds to the theoretical synthesis of sustainability competence, cultural sustainability, intangible heritage transmission, music education, and digital cultural participation outlined in Section 2.4.

The five retained dimensions reflect different but related aspects of guzheng cultural sustainability competence. The positive correlations among the dimensions suggest that they share a common orientation toward cultural learning. However, the correlations were not so high as to suggest redundancy. The strongest association was found between heritage identity and embodied performance and aesthetic literacy, which is reasonable in guzheng education. Students who more strongly recognize the cultural value of the guzheng may also be more likely to connect technique, repertoire, style, and aesthetic judgement.

Creative adaptation was moderately associated with both digital transmission and community engagement. This pattern suggests that innovation readiness may be connected with outward-facing cultural communication. Students who are more open to creative adaptation may also be more prepared to use digital media or public contexts to communicate guzheng culture. This does not imply causality or developmental sequencing. Rather, the observed correlations indicate that higher self-reported scores in creative adaptation, digital communication, and community engagement tended to co-occur within this sample.

It is worth noting that this weak relationship between digital transmission and heritage identity exists. Readiness to communicate digitally cannot be directly derived based on the presence of a powerful cultural identity. There are students who can appreciate guzheng as heritage yet have no confidence, experience or interest in communicating it using virtual media or other types of public facing formats. It is therefore justified to view digital transmission as an isolated competence dimension and not presuppose that it is inherently incorporated into the traditional performance training.

### 5.4 Heritage identity and embodied performance literacy as higher-scoring dimensions

The students obtained the best results in heritage identity and cultural responsibility and in embodied performance and aesthetic literacy. Such a finding is consistent with the educational environment of university guzheng programs. Most students come to university after years of previous training where the study of technique, repertoire, tone production, stylistic interpretation, and performance evaluation are often used to structure the learning. These experiences might be reasons that can account for why students indicated a high level of knowledge about the cultural heritage of the guzheng and great assurance in linking performance technique with aesthetic meaning.

The high heritage identity indicates that a large number of students did not see the guzheng learning as merely a technical task. Instead, they were more inclined to see the instrument as part of the Chinese traditional music culture and as an act of culture transmission. It is in line with the literature that the contemporary Chinese zheng has been evolving under professionalization and modernization maintaining close ties with cultural identity and historical continuity (Sun, 2015; Silverberg, 2022; Han, 2013).

The relatively high score for embodied performance and aesthetic literacy is consistent with the central place of bodily practice, listening, repertoire study, stylistic interpretation, and aesthetic judgment in music education (Lau, 2008; Schippers, 2010; Elliott and Silverman, 2015). However, the present cross-sectional data do not allow us to attribute these scores to the effectiveness of particular university programs or prior training experiences. The observed pattern is more cautiously interpreted as being consistent with the prominence of heritage- and performance-oriented learning within the educational context represented by this sample.

These relatively higher scores should also not be interpreted as evidence that the corresponding competencies are fully developed. They represent students' self-reported perceptions rather than direct assessments of performance or cultural understanding. Their contrast with the lower scores observed for digital communication and community-oriented participation may therefore be useful for identifying questions for subsequent research rather than for making definitive judgments about program strengths and weaknesses.

### 5.5 Digital transmission and community engagement as lower-scoring dimensions

The mean of digital transmission and public communication was the least and the standard deviation was the biggest. It indicates that students are likely to differ significantly on their preparedness to utilize digital platforms or public communication to disseminate guzheng culture. There might be some students who are already at ease with the idea of short videos, online performances or the concept of sharing knowledge digitally. The other group may have a little experience, low confidence, or even low interest in these activities.

Digital transmission and public communication had the lowest mean score and the largest standard deviation among the five dimensions. This pattern indicates greater variability in students' self-reported readiness for digital and public communication. However, the data do not establish whether the lower ratings resulted from limited instructional opportunities, lower confidence, less experience, lower interest, or other factors not measured in the present study.

Community engagement and sustainable career readiness also received lower ratings than heritage identity and embodied performance literacy. This descriptive contrast is consistent with the participant-background data, which showed that public performance experience was more common than participation in community- or heritage-oriented activities. Nevertheless, the cross-sectional design does not permit the conclusion that curriculum structure caused these differences.

If future research confirms these patterns in larger samples and demonstrates links with relevant learning experiences, digital cultural communication and community-oriented participation may represent useful areas for pedagogical exploration. Possible approaches could include audience-oriented cultural interpretation, digital communication activities, community outreach, or career-related learning. These possibilities should be treated as hypotheses for educational practice rather than interventions shown to be effective by the present study.

### 5.6 The boundary between creative adaptation and digital competence

The removal of CA4 and DT4 provides useful information about the boundary between creative adaptation and digital competence. CA4 referred to confidence in designing cross-genre guzheng performances for modern audiences. Although this item was theoretically assigned to creative adaptation and repertoire innovation, it loaded more strongly on the digital transmission and public communication factor in the initial solution. This suggests that students may have interpreted cross-genre design for modern audiences as a communication-oriented or media-oriented practice rather than as a general form of creative adaptation readiness.

DT4 referred to familiarity with digital or AI-based tools for guzheng learning, performance, or communication. Although this item was theoretically assigned to digital transmission and public communication, it showed a cross-domain pattern involving creative adaptation and digital transmission. This suggests that advanced digital or AI-based tool use may not function as a basic indicator of public communication readiness. Instead, students may associate it with innovation, experimentation, or specialized creative practice.

These two items should not be regarded simply as weak items. Rather, they point to a possible boundary area between creative production and digital-cultural communication. Future studies may develop additional items to examine advanced creative-digital competence, especially in relation to cross-media performance, AI-assisted music learning, and digital heritage communication. For the present scale, however, removing CA4 and DT4 produced a clearer and more interpretable 18-item structure.

### 5.7 Exploratory learner profiles and possible educational considerations

The exploratory four-cluster solution provides a descriptive illustration of how different subscale-score patterns co-occurred in the present sample. The profile labels are therefore analytical descriptions rather than fixed categories of students.

The profile labeled balanced cultural competence learners showed relatively high scores across all five dimensions. The tradition-oriented performer profile combined relatively high heritage identity and embodied performance literacy with lower digital communication and community engagement scores. The innovation-ready communicator profile showed comparatively higher creative-adaptation and digital-transmission scores, whereas the skill-focused profile showed lower scores across several dimensions. These patterns describe relative differences within the selected cluster solution and should not be interpreted as objective judgments of student proficiency.

The profiles may nevertheless generate hypotheses for future educational research. For example, subsequent studies could examine whether students with different score patterns respond differently to activities involving cultural interpretation, digital communication, community participation, or repertoire innovation. Such possibilities require direct empirical testing. The present cluster analysis alone does not demonstrate that a particular profile needs, or would benefit from, a specific form of instructional support. Illustrative educational considerations associated with the exploratory learner profiles are summarized in Table 9.

**Table 9:** Illustrative educational considerations associated with the exploratory profile patterns.

Exploratory profile	Observed relative score pattern	Possible area for further investigation	Illustrative educational consideration
Balanced cultural competence learners	Relatively high self-reported scores across all five dimensions	Relationships between multidimensional self-perceptions and cultural participation	Integrated heritage interpretation, interdisciplinary projects, and community-based cultural activities
Tradition-oriented performers	Higher heritage identity and embodied performance scores, with comparatively lower digital communication scores	Factors associated with digital and public communication readiness	Digital storytelling, audience-oriented cultural interpretation, and public communication activities
Innovation-ready communicators	Relatively higher creative adaptation and digital communication scores, with lower community engagement scores	Relationships between creative adaptation, cultural interpretation, and community participation	Traditional and contemporary repertoire comparison, culturally informed innovation, and community outreach
Skill-focused learners with limited cultural engagement	Relatively lower scores across the five dimensions, particularly creative adaptation, digital communication, and community engagement	Learning experiences associated with cultural engagement and participation readiness	Heritage interpretation activities, community-based learning, and exploration of cultural career pathways

The educational considerations are illustrative possibilities derived from descriptive cluster patterns. They do not represent individualized diagnoses, causal conclusions, or empirically tested intervention recommendations. The four-cluster solution was exploratory and requires replication in independent samples before profile-specific educational applications can be evaluated.

### 5.8 . Potential educational implications

The present findings do not test the effectiveness of any instructional approach. Nevertheless, the observed score patterns may help identify topics for future pedagogical investigation in culturally embedded higher arts education. In particular, the contrast among heritage-related, performance-related, digital, and community-oriented dimensions suggests that cultural sustainability may be examined as a broader educational framework alongside conventional technical and repertoire-based learning.

For example, future studies could evaluate whether incorporating cultural interpretation into performance learning helps students connect repertoire with historical context, regional style, aesthetic meaning, and heritage significance. Digital communication could also be studied as a possible learning context through activities such as interpretive videos, lecture-performances, digital program notes, or educational materials for non-specialist audiences. Similarly, community-based cultural practice could be examined through school outreach, museums, cultural centers, or heritage-related activities.

Creative adaptation and career-related learning represent additional areas for investigation. Comparative discussion of traditional and contemporary repertoire may provide one way of examining how students negotiate continuity and innovation, while exposure to performance, teaching, cultural communication, community education, and heritage-related work may help researchers explore how students perceive broader professional pathways. These suggestions are not effects demonstrated by the present cross-sectional study. They are possible directions for subsequent educational research derived from the descriptive patterns observed here.

### 5.9 Limitations and future research

Several limitations should be acknowledged. First, the study used a regional multi-institutional sample from Northeast China. Although the sample included five higher education institutions with different institutional profiles, it was not nationally representative. The findings should therefore not be generalized to all guzheng students in China. Future research should test the GCSCS with broader samples from different regions and institutional contexts.

Second, all substantive variables in this study were based on self-report. The GCSCS therefore captures students' perceptions of their understanding, capability, judgment, and readiness rather than independently demonstrated competence. Higher or lower scores should not be interpreted as direct evidence of actual performance quality, communication effectiveness, creative achievement, or community participation. Future validation should combine GCSCS scores with external criteria such as performance assessment, portfolio evidence, digital communication products, or other independent indicators.

Third, the study provided preliminary validation evidence rather than full validation. The EFA results were interpretable, and the reliability indices were acceptable, but confirmatory factor analysis should be conducted with an independent sample. Future studies should also examine convergent, discriminant, and criterion-related validity. Measurement invariance across gender, educational level, institution type, and region should also be tested when larger samples are available.

Fourth, the cluster analysis was exploratory and the separation among solutions was modest. The four-cluster solution was selected for substantive interpretability and acceptable group sizes rather than because it was statistically optimal across all diagnostic indices. In fact, the three-cluster solution produced a marginally higher silhouette coefficient (0.229) than the four-cluster solution (0.223). The resulting profiles should therefore be interpreted as provisional descriptive patterns, not as stable or naturally occurring categories of guzheng students. Replication with larger independent samples and alternative person-centered methods is needed before stronger claims about learner-profile structure can be made.

Fifth, CA4 and DT4 were not retained in the final 18-item scale, but their loading patterns suggest an important area for future research. Advanced creative-digital competence may be a meaningful construct in guzheng education, especially in relation to cross-genre performance, AI-assisted music learning, and digital heritage communication. Future studies may develop additional items to examine this boundary area more precisely.

Sixth, several GCSCS items were phrased in terms of beliefs, willingness, interests, responsibility, or readiness rather than direct perceived capability. Although these indicators are relevant to culturally sustainable participation, they are not equivalent to perceived ability and may blur the boundary between competence-related self-perceptions and dispositional orientations. Future scale refinement should therefore develop more explicitly capability-based items within each dimension and examine, in an independent sample, whether perceived capability and dispositional readiness represent distinguishable but related components of the broader construct.

Despite these limitations, the study provides initial evidence that the GCSCS can be used as a domain-specific instrument for examining students' perceived cultural sustainability competence in university guzheng education. It also shows that guzheng students differ in meaningful ways across heritage identity, embodied performance literacy, creative adaptation, digital communication, and community engagement. These findings may support future research on culturally embedded music learning, learner profiling, and differentiated educational support in higher arts education.

Future studies may also examine the broader psychosocial correlates of cultural sustainability-related self-perceptions. Variables such as self-efficacy, perceived social support, psychological resilience, subjective well-being, and community participation could provide useful external criteria for examining the educational and psychological relevance of the GCSCS. Such research would be needed before any claims could be made about relationships between culturally engaged arts learning and students' psychosocial adjustment or wellbeing.

## 6 Conclusions

This study developed and preliminarily evaluated the Guzheng Cultural Sustainability Competence Scale (GCSCS) as a domain-specific self-report instrument for examining multidimensional student perceptions related to culturally sustainable guzheng learning. In a regional sample of 168 students from five higher education institutions, the final 18-item version showed an interpretable five-factor structure and acceptable internal consistency across heritage identity and cultural responsibility, embodied performance and aesthetic literacy, creative adaptation and repertoire innovation, digital transmission and public communication, and community engagement and sustainable career readiness.

The descriptive results showed higher self-reported scores for heritage identity and embodied performance literacy and lower scores for digital communication and community-oriented dimensions. These differences should be interpreted as relative score patterns within the present sample rather than as objective evidence of stronger or weaker competence. Similarly, the exploratory cluster analysis yielded four descriptive profile patterns, but the modest separation among candidate solutions means that these profiles should not be treated as stable learner categories.

The study therefore provides preliminary psychometric evidence for a theory-informed, domain-specific framework linking cultural continuity, embodied artistic practice, responsible adaptation, public communication, and community participation. The findings may help frame questions for future research in culturally embedded higher arts education, but they do not demonstrate the effectiveness of particular curricula or instructional strategies.

Further research should test the factor structure in larger and more diverse independent samples and examine convergent, discriminant, and criterion-related validity. External assessments of performance, communication, creative work, or cultural participation would also help clarify the relationship between students' self-perceptions and demonstrated competence. Longitudinal or intervention-based designs would be needed to examine developmental or causal questions. Future studies may additionally investigate whether cultural sustainability-related self-perceptions are associated with broader psychosocial resources and outcomes, including self-efficacy, social support, psychological resilience, subjective well-being, and community participation.

## Data Availability

The anonymized participant-level data are not publicly available due to participant confidentiality and data protection considerations. Requests for access may be directed to the corresponding authors and will be considered upon reasonable request, subject to applicable institutional and participant-protection requirements.
