# Supplementary material for: Measuring student-perceived cultural sustainability competence in guzheng education: development and preliminary validation of the GCSCS
Source: Front Psychol. 2026 Oct 28;17:1900263. doi: 10.3389/fpsyg.2026.1900263 (PMC13752120; doi:10.3389/fpsyg.2026.1900263)
Supplement: Supplementary file 1 [file Supplementary_File_1.docx]

**Appendix A. Initial Survey Instrument for the Guzheng Cultural Sustainability Competence Scale**

Instruction: Please indicate the extent to which you agree with each statement based on your guzheng learning experience.

Response format: 1 = Strongly disagree; 2 = Disagree; 3 = Neutral; 4 = Agree; 5 = Strongly agree.

A1. Heritage Identity and Cultural Responsibility

HI1. I understand guzheng performance as a form of cultural heritage rather than only a technical skill.

HI2. Learning guzheng strengthens my understanding of Chinese traditional music culture.

HI3. I believe that guzheng students have a responsibility to contribute to the transmission of traditional music.

HI4. I can explain the cultural meaning of selected guzheng works to audiences or learners.

A2. Embodied Performance and Aesthetic Literacy

EP1. I can connect finger technique, tone production, and musical expression in guzheng performance.

EP2. I pay attention to stylistic differences among guzheng schools, repertoires, and historical contexts.

EP3. I understand that guzheng learning involves embodied practice, listening, imitation, and long-term aesthetic cultivation.

EP4. I can interpret guzheng works through both technical control and cultural understanding.

A3. Creative Adaptation and Repertoire Innovation

CA1. I support the creative adaptation of guzheng music when traditional aesthetic principles are respected.

CA2. I am willing to explore contemporary compositions or interdisciplinary forms involving guzheng.

CA3. I can distinguish meaningful innovation from superficial commercialization in guzheng performance.

CA4. I am confident in designing cross-genre guzheng performances for modern audiences.

A4. Digital Transmission and Public Communication

DT1. I am willing to use digital platforms to disseminate guzheng culture to wider audiences.

DT2. I can present guzheng-related knowledge in a way that is understandable to non-specialist audiences.

DT3. I believe online performance, short videos, or digital archives can support the sustainable transmission of guzheng culture.

DT4. I am familiar with using digital or AI-based tools to support guzheng learning, performance, or communication.

A5. Community Engagement and Sustainable Career Readiness

CE1. I am willing to participate in community-based guzheng education or cultural outreach activities.

CE2. I believe guzheng learning can support my long-term professional development.

CE3. I am interested in connecting guzheng performance with public education, heritage transmission, or cultural services.

CE4. I can see possible career pathways that combine guzheng performance, teaching, cultural communication, and social engagement.

A6. Background Information

B1. Gender: Male / Female / Prefer not to say

B2. Age: ____

B3. Undergraduate / Postgraduate / Other

B4. Year of study: Year 1 / Year 2 / Year 3 / Year 4 / Postgraduate

B5. Years of guzheng learning: ____ years

B6. Average weekly practice time: ____ hours

B7. Have you participated in guzheng competitions? Yes / No

B8. Have you participated in public performances? Yes / No

B9. Have you participated in community, school, or heritage-related guzheng activities? Yes / No

B10. Have you used digital platforms to share guzheng performance or knowledge? Yes / No

Note: The questionnaire was administered in Chinese. The English version is provided for manuscript reporting. CA4 and DT4 were included in the initial survey but were not retained in the final 18-item scale after exploratory factor analysis.The wording shown above reflects the questionnaire administered during data collection. The items were not retrospectively rewritten after data collection. As clarified in the revised manuscript, some items represent perceived capability, whereas others represent understanding, judgment, or readiness-related dispositions.

**Supplementary Table S1. Expert panel for content validity appraisal.**

| Expert code | Area of expertise | Relevant background |
| --- | --- | --- |
| E1 | Guzheng performance | University-level guzheng teaching and performance |
| E2 | Guzheng pedagogy | Traditional instrumental music education |
| E3 | Music education | Higher music education and arts education research |
| E4 | Educational measurement | Scale development and educational assessment |
| E5 | Traditional Chinese music culture | Chinese traditional music and cultural heritage studies |

Note: Expert names were not reported to protect confidentiality. The expert appraisal focused on item relevance, clarity, dimensional fit, and cultural appropriateness.

**Supplementary Table S2. Item-level screening results for the initial 20-item GCSCS.**

| **Item** | **Dimension** | **Mean** | **SD** | **Corrected item-total correlation** | **Cronbach’s α if item deleted** | **Retention decision** |
| --- | --- | --- | --- | --- | --- | --- |
| HI1 | Heritage identity and cultural responsibility | 4.29 | 0.67 | 0.528 | 0.898 | Retained in the final scale |
| HI2 | Heritage identity and cultural responsibility | 4.33 | 0.62 | 0.405 | 0.901 | Retained in the final scale |
| HI3 | Heritage identity and cultural responsibility | 4.15 | 0.67 | 0.571 | 0.897 | Retained in the final scale |
| HI4 | Heritage identity and cultural responsibility | 4.18 | 0.64 | 0.446 | 0.900 | Retained in the final scale |
| EP1 | Embodied performance and aesthetic literacy | 4.14 | 0.68 | 0.472 | 0.899 | Retained in the final scale |
| EP2 | Embodied performance and aesthetic literacy | 4.17 | 0.68 | 0.546 | 0.898 | Retained in the final scale |
| EP3 | Embodied performance and aesthetic literacy | 4.12 | 0.65 | 0.434 | 0.900 | Retained in the final scale |
| EP4 | Embodied performance and aesthetic literacy | 4.04 | 0.68 | 0.462 | 0.900 | Retained in the final scale |
| CA1 | Creative adaptation and repertoire innovation | 3.79 | 0.85 | 0.588 | 0.896 | Retained in the final scale |
| CA2 | Creative adaptation and repertoire innovation | 3.67 | 0.74 | 0.571 | 0.897 | Retained in the final scale |
| CA3 | Creative adaptation and repertoire innovation | 3.64 | 0.85 | 0.563 | 0.897 | Retained in the final scale |
| CA4 | Creative adaptation and repertoire innovation | 3.57 | 0.93 | 0.509 | 0.899 | Not retained after EFA |
| DT1 | Digital transmission and public communication | 3.51 | 0.97 | 0.504 | 0.899 | Retained in the final scale |
| DT2 | Digital transmission and public communication | 3.54 | 0.95 | 0.518 | 0.899 | Retained in the final scale |
| DT3 | Digital transmission and public communication | 3.47 | 1.00 | 0.643 | 0.895 | Retained in the final scale |
| DT4 | Digital transmission and public communication | 3.33 | 1.00 | 0.575 | 0.897 | Not retained after EFA |
| CE1 | Community engagement and sustainable career readiness | 3.66 | 0.90 | 0.538 | 0.898 | Retained in the final scale |
| CE2 | Community engagement and sustainable career readiness | 3.62 | 0.82 | 0.578 | 0.897 | Retained in the final scale |
| CE3 | Community engagement and sustainable career readiness | 3.51 | 0.84 | 0.594 | 0.896 | Retained in the final scale |
| CE4 | Community engagement and sustainable career readiness | 3.60 | 0.88 | 0.663 | 0.894 | Retained in the final scale |

Note: Item screening was used together with exploratory factor analysis and theoretical interpretability. CA4 and DT4 were not removed because of poor item-total correlations; they were removed because their factor loading patterns did not correspond clearly to their theoretically assigned dimensions.

**Supplementary Table S3. Full pattern matrix for the initial 20-item GCSCS.**

| **Item** | **Theoretical dimension** | **HI** | **EP** | **CA** | **DT** | **CE** | **Primary factor** | **Primary loading** | **Communality** | **Decision** |
| --- | --- | --- | --- | --- | --- | --- | --- | --- | --- | --- |
| HI1 | Heritage identity and cultural responsibility | 0.710 | -0.053 | 0.095 | 0.044 | -0.020 | HI | 0.710 | 0.528 | Retained |
| HI2 | Heritage identity and cultural responsibility | 0.563 | 0.057 | -0.024 | -0.067 | 0.081 | HI | 0.563 | 0.389 | Retained |
| HI3 | Heritage identity and cultural responsibility | 0.559 | 0.059 | 0.020 | 0.055 | 0.141 | HI | 0.559 | 0.512 | Retained |
| HI4 | Heritage identity and cultural responsibility | 0.586 | 0.120 | -0.018 | -0.003 | 0.003 | HI | 0.586 | 0.429 | Retained |
| EP1 | Embodied performance and aesthetic literacy | 0.113 | 0.611 | 0.031 | 0.083 | -0.051 | EP | 0.611 | 0.477 | Retained |
| EP2 | Embodied performance and aesthetic literacy | 0.355 | 0.418 | 0.015 | 0.054 | 0.019 | EP | 0.418 | 0.513 | Retained |
| EP3 | Embodied performance and aesthetic literacy | 0.114 | 0.537 | 0.080 | -0.071 | 0.048 | EP | 0.537 | 0.422 | Retained |
| EP4 | Embodied performance and aesthetic literacy | -0.048 | 0.739 | 0.007 | -0.015 | 0.107 | EP | 0.739 | 0.582 | Retained |
| CA1 | Creative adaptation and repertoire innovation | 0.035 | 0.100 | 0.709 | 0.073 | -0.088 | CA | 0.709 | 0.583 | Retained |
| CA2 | Creative adaptation and repertoire innovation | 0.031 | 0.071 | 0.533 | 0.091 | 0.059 | CA | 0.533 | 0.445 | Retained |
| CA3 | Creative adaptation and repertoire innovation | 0.050 | -0.051 | 0.715 | -0.081 | 0.119 | CA | 0.715 | 0.549 | Retained |
| CA4 | Creative adaptation and repertoire innovation | 0.059 | 0.006 | 0.129 | 0.461 | 0.066 | DT | 0.461 | 0.368 | Not retained |
| DT1 | Digital transmission and public communication | -0.062 | 0.012 | 0.050 | 0.837 | -0.070 | DT | 0.837 | 0.694 | Retained |
| DT2 | Digital transmission and public communication | 0.004 | 0.053 | -0.004 | 0.742 | -0.019 | DT | 0.742 | 0.556 | Retained |
| DT3 | Digital transmission and public communication | 0.091 | -0.042 | -0.039 | 0.804 | 0.125 | DT | 0.804 | 0.734 | Retained |
| DT4 | Digital transmission and public communication | -0.051 | -0.074 | 0.432 | 0.348 | 0.128 | CA | 0.432 | 0.536 | Not retained |
| CE1 | Community engagement and sustainable career readiness | -0.018 | 0.055 | 0.003 | 0.017 | 0.680 | CE | 0.680 | 0.495 | Retained |
| CE2 | Community engagement and sustainable career readiness | 0.113 | -0.066 | 0.002 | -0.008 | 0.738 | CE | 0.738 | 0.604 | Retained |
| CE3 | Community engagement and sustainable career readiness | -0.015 | 0.074 | 0.177 | 0.035 | 0.534 | CE | 0.534 | 0.472 | Retained |
| CE4 | Community engagement and sustainable career readiness | 0.007 | 0.184 | 0.033 | 0.095 | 0.611 | CE | 0.611 | 0.604 | Retained |

Note: HI = heritage identity and cultural responsibility; EP = embodied performance and aesthetic literacy; CA = creative adaptation and repertoire innovation; DT = digital transmission and public communication; CE = community engagement and sustainable career readiness. CA4 was theoretically assigned to CA but loaded most strongly on the DT factor. DT4 was theoretically assigned to DT but loaded most strongly on the CA factor and also showed a secondary loading on DT. These loading patterns indicated construct-boundary problems and supported their removal from the final 18-item scale.

**Supplementary Table S4. Cluster solution diagnostics.**

| **k** | **Method** | **Cluster sizes** | **Minimum group size** | **Silhouette coefficient** | **Within-cluster SSE / inertia** | **Mean centroid distance** | **Minimum centroid distance** | **Interpretability** | **Main issue** | **Decision** |
| --- | --- | --- | --- | --- | --- | --- | --- | --- | --- | --- |
| 3 | k-means on z-standardized HI, EP, CA, DT, CE scores | 42, 82, 44 | 42 | 0.229 | 439.771 | 2.662 | 1.960 | Moderate; separates low-engagement and high-competence groups, but merges tradition-oriented and balanced learners | Too coarse for learner-profile interpretation | Not selected |
| 4 | k-means on z-standardized HI, EP, CA, DT, CE scores | 31, 44, 48, 45 | 31 | 0.223 | 375.515 | 2.713 | 1.915 | High; yields four pedagogically interpretable profiles with acceptable minimum group size | Silhouette is close to the three-cluster solution, so interpretation should remain preliminary | Selected |
| 5 | k-means on z-standardized HI, EP, CA, DT, CE scores | 37, 26, 33, 31, 41 | 26 | 0.192 | 341.672 | 2.683 | 1.707 | Lower; divides high-competence learners into smaller subgroups | Less parsimonious and includes a smaller group | Not selected |

Note: The diagnostic indices were considered jointly and were not treated as formal tests proving a unique optimal cluster structure. Although the three-cluster solution had a marginally higher silhouette coefficient than the four-cluster solution, the four-cluster solution was retained for exploratory interpretation because it preserved additional substantively interpretable score-pattern distinctions while maintaining acceptable group sizes. The profiles should therefore be regarded as provisional and require replication in independent samples.
